# MEG Analysis of Neural Interactions in Attention-Deficit/Hyperactivity Disorder

**DOI:** 10.1155/2016/8450241

**Published:** 2016-03-29

**Authors:** Amine Khadmaoui, Carlos Gómez, Jesús Poza, Alejandro Bachiller, Alberto Fernández, Javier Quintero, Roberto Hornero

**Affiliations:** ^1^Biomedical Engineering Group, E.T.S. Ingenieros de Telecomunicación, Universidad de Valladolid, Campus Miguel Delibes, Paseo Belén 15, 47011 Valladolid, Spain; ^2^IMUVA, Instituto de Investigación en Matemáticas, Universidad de Valladolid, Campus Miguel Delibes, Paseo Belén 7, 47011 Valladolid, Spain; ^3^Department of Psychiatry, Facultad de Medicina, Universidad Complutense de Madrid, Ciudad Universitaria, 28040 Madrid, Spain; ^4^Laboratory of Cognitive and Computational Neuroscience, Centro de Tecnología Biomédica, Campus Montegancedo, Pozuelo de Alarcón, 28223 Madrid, Spain; ^5^Psychiatry Service, Hospital Infanta Leonor, Avenida Gran Vía del Este 80, 28031 Madrid, Spain

## Abstract

The aim of the present study was to explore the interchannel relationships of resting-state brain activity in patients with attention-deficit/hyperactivity disorder (ADHD), one of the most common mental disorders that develop in children. Magnetoencephalographic (MEG) signals were recorded using a 148-channel whole-head magnetometer in 13 patients with ADHD (range: 8–12 years) and 14 control subjects (range: 8–13 years). Three complementary measures (coherence, phase-locking value, and Euclidean distance) were calculated in the conventional MEG frequency bands: delta, theta, alpha, beta, and gamma. Our results showed that the interactions among MEG channels are higher for ADHD patients than for control subjects in all frequency bands. Statistically significant differences were observed for short-distance values within right-anterior and central regions, especially at delta, beta, and gamma-frequency bands (*p* < 0.05; Mann-Whitney *U* test with false discovery rate correction). These frequency bands also showed statistically significant differences in long-distance interactions, mainly among anterior and central regions, as well as among anterior, central, and other areas. These differences might reflect alterations during brain development in children with ADHD. Our results support the role of frontal abnormalities in ADHD pathophysiology, which may reflect a delay in cortical maturation in the frontal cortex.

## 1. Introduction

Attention-deficit/hyperactivity disorder (ADHD) is a persistent neuropsychiatric disorder, which changes with development from childhood through adulthood [1]. It is characterized by excessive impulsivity and hyperactivity and/or by difficulty in maintaining attention. These features predispose the children to psychiatric and social pathology, in addition to academic dysfunctions or chaotic interpersonal relationships in later life [1]. Prevalence rates of ADHD depend on distinct factors, such as the diagnostic criteria and the sampled population. However, the prevalence rate was placed by several studies to be about 4–6% [2, 3], making this disorder commonly diagnosed in childhood. Despite its clear medical, social, and familial relevance, the nature of this dysfunction is not entirely understood and there is no neurobiological marker defined for it. Therefore, diagnosis relies exclusively on clinical criteria, such as Diagnostic and Statistical Manual of Mental Disorders (DSM) and International Statistical Classification of Diseases (ICD). Nevertheless, clinicians still need to collect data from informants (parents and teachers) and follow developmental variations in symptom expression [4].

In the recent years, neuroimaging research has evolved rapidly, providing several ways to examine the pathophysiology of ADHD and the biological effects of medical treatments. The earliest neuroimaging studies used single-photon emission computed tomography (SPECT) and positron-emission tomography (PET), showing frontostriatal abnormalities in ADHD [5, 6]. Later, these techniques have been replaced by functional magnetic resonance imaging (fMRI), which offers better spatial and temporal resolution for functional researches [7]. Most fMRI studies revealed that ADHD might be related to dorsal anterior cingulate cortex dysfunction, which has been shown to be essential in cognition, attention, and decision making [8, 9]. In another study, ADHD was defined as a disorder characterized by a delay of cortical maturation, which was most prominent in prefrontal regions [10]. Additionally, Bush et al. [7] reviewed several functional neuroimaging studies in ADHD and concluded that patients showed a consistent pattern of frontal dysfunction in the brain. In sum, neuroimaging techniques provide extensive evidence for brain dysfunction in ADHD.

To understand dynamic cognitive processes, neurophysiological techniques, such as electroencephalography (EEG) and magnetoencephalography (MEG), are required. Both allow acquiring neural activity with higher temporal resolution than SPECT, PET, and fMRI [11]. EEG and MEG are completely noninvasive and record the electromagnetic oscillations produced by cerebral activity directly, without the need to interpret it in terms of proxy measure [11, 12]. Nevertheless, there are some differences between these techniques. While electric fields are strongly influenced by several factors, such as distance between sensors or electrode location, magnetic fields are reference-free and less affected by distortions produced by the resistive properties of the skull and the scalp [11]. However, MEG equipment is characterized by limited availability and high costs, in comparison to EEG devices [13]. Several studies evidenced the utility of EEG/MEG analysis to evaluate the brain activity in ADHD. Spectral analysis revealed that ADHD patients showed significantly higher theta relative power and lower beta relative power, along with higher theta/alpha and theta/beta ratios [14]. Likewise, recent studies suggested that nonlinear measures can complement spectral analysis in order to understand brain dynamics in ADHD [15]. For instance, MEG data were analyzed by using Lempel-Ziv complexity [16] and fuzzy entropy [15]. They revealed that brain activity is less complex and more regular in ADHD patients compared to controls. In sum, accumulating evidence suggests that EEG and MEG are useful to explore the neurophysiological substrate of neural dysfunction in ADHD. However, further research is necessary to characterize the neural dynamics associated with this disorder.

All the aforementioned research works analyzed EEG/MEG activity by studying local activation patterns (i.e., neural activity recorded from each channel is evaluated independently). However, several authors considered that higher brain functions depend on a balance between local specialization and global integration of brain processes [17]. Therefore, a better comprehension of the brain as a complex structural and functional network is needed [18]. To achieve this, it is important to study how neural couplings are carried out (for a review about brain connectivity, see Friston [19] and/or Sakkalis [20]). Although connectivity patterns have been widely investigated in other brain disorders, only a few studies have focused on ADHD [21–24]. For instance, Clarke et al. [21] showed that ADHD children have an underlying brain dysfunction in the frontal lobes, by means of mean square EEG coherence. Other authors computed MEG phase coherence during an auditory attention task, revealing hyperconnectivity in the high frequency range in adults with ADHD compared to controls [22].

Most of the previous coupling studies used a single measure, mainly coherence, to analyze brain dynamics in ADHD. In this study, three complementary measures have been applied in order to get a comprehensive characterization of the interchannel relationships in ADHD. To begin with, coherence (COH) is a normalized linear measure widely used to explore connectivity at a particular frequency. In essence, it quantifies linear correlations in the frequency domain [25]. Secondly, phase-locking value (PLV) is a synchronization measure that identifies whether frequency specific transient phase locking exists [26]. Finally, Euclidean distance (ED) is a similarity measure, which has been proposed to assess the statistical distance between probability distributions [27, 28].

The aim of our study is to provide further insights into the underlying brain dynamics associated with ADHD. For this purpose, COH, PLV, and ED were calculated in the conventional MEG frequency bands (*δ*, *θ*, *α*, *β*, and *γ*). We attempt to address the following research questions: (i) How does ADHD affect the neural interaction patterns? (ii) Can our methodology reflect the regional abnormalities of ADHD? (iii) Can the proposed measures provide complementary information about neural dynamics?

## 2. Materials and Methods

### 2.1. MEG Recording

Brain magnetic fields were acquired from each participant with a 148-channel whole-head magnetometer (MAGNES 2500 WH, 4D Neuroimaging) located in a magnetically shielded room at the MEG Center Dr. Pérez-Modrego (Spain). During MEG recording, subjects were lying comfortably on a patient bed, in a relaxed state, and with their eyes closed. They were instructed to stay awake and to avoid eye and head movements in order to reduce the presence of artifacts in the recordings. Their behavior was controlled during the recording procedure by means of a video-camera. Additionally, technicians may communicate with children during MEG acquisition using a loud-speaking intercom. Participants included in the final sample did not exhibit significant difficulties in maintaining their immobility.

For each subject, five minutes of MEG data was acquired at a sampling frequency of 678.17 Hz. A process of downsampling by a factor of four was carried out, resulting in a sampling rate of 169.55 Hz. Data were then digitally filtered using a 1–65 Hz band-pass filter and a 50 Hz notch filter. To minimize the presence of oculographic, cardiographic, and myographic artifacts, both visual inspection and independent component analysis (ICA) were performed [15]. Finally, artifact-free epochs of 848 samples (5 s) were selected for further analyses.

### 2.2. Subjects

In this study, MEG data were acquired from 27 subjects. The clinical group comprised 13 children with ADHD (age = 9.5 ± 1.3 years, mean ± standard deviation, SD; range 8–12 years). Inclusion criteria included a full DSM-IV (DSM, Fourth Edition) diagnosis of ADHD combined type with associated impairment in at least two settings and Conners' Parent Rating Scale (CPRS) hyperactivity rating greater than two SD above age- and sex-specific means [29]. The DSM-IV diagnosis of ADHD was based on the parent version of the Diagnostic Interview for Children and Adolescents [30]. ADHD patients had never used any psychoactive drug or received any psychoactive therapy.

The control group was formed by 14 healthy children (10.4 ± 1.5 years, mean ± SD; range 8–13 years) without past or present neurological disorders. There were not significant differences (*p* values > 0.05, Mann-Whitney *U* test) between ADHD patients and control subjects in terms of age and years of education (6.8 ± 1.2 years in ADHD patients and 7.3 ± 1.4 years in controls; mean ± SD) and they all were strictly right-handed. Written informed consent and assent to participate in the study were obtained from parents and children, respectively. The Institutional Review Board approved the research protocol.

### 2.3. Neural Interaction Measures

The study of neural interactions seeks to understand how different regions of the brain cortex communicate with each other. In this research, we focused on three complementary points of view, which have been developed to analyze these neural connections: COH, PLV, and ED.

#### 2.3.1. Coherence (COH)

COH is a normalized linear measure that provides information about the degree of coupling between two signals within a given frequency band [25, 31]. It has been commonly used in neuroscience to evaluate the correlation among the neurophysiological signals measured at different sensors [32]. This measure has been previously used to analyze the brain background activity in ADHD [21, 23, 24].

In particular, mean square coherence has been calculated. It is a real-valued function defined as the square cross-spectrum normalized by the product of the two power spectra. Having two MEG channels, *x*(*t*) and *y*(*t*), COH can be calculated as follows [25]:

(1) COHxyband=∑f∈bandSxyf2Sxxf·Syyf,band=δ,θ,α,β,γ,

where *S*_*xy*_(*f*) is the cross-power spectral density and *S*_*xx*_(*f*) and *S*_*yy*_(*f*) are the respective autopower spectral densities [25]. COH values range from 0 to 1. A value of 0 indicates no linear dependence between signals, whereas a value of 1 is obtained when they are perfectly coupled.

#### 2.3.2. Phase-Locking Value (PLV)

PLV is a highly sensitive measure of neural synchronization that specifically identifies whether frequency transient phase locking exists. It quantifies the relationship between the phases of *x*(*t*) and *y*(*t*), while their amplitudes could be uncorrelated [33].

To calculate PLV, firstly it is necessary to constrain the spectrum by filtering on the frequency band of interest. Then, Hilbert transform was used to extract the instantaneous phase, *φ*_*x*_^band^(*t*) and *φ*_*y*_^band^(*t*), from the two signals, *x*(*t*) and *y*(*t*), respectively. Thus, the instantaneous phase difference for epoch *n* was obtained as follows [26]:

(2) $$\begin{array}{c} \Delta \varphi_{xy}^{\text{band}}\left(\;t,n\;\right)=\varphi_{x}^{\text{band}}\left(\;t,n\;\right)-\varphi_{y}^{\text{band}}\left(\;t,n\;\right). \end{array}$$

Finally, PLV was calculated as the variability of the instantaneous phase difference:

(3) $$\begin{array}{c} {\text{PLV}}_{xy}^{\text{band}}=\frac{1}{N}\left|\;\overset{N}{\underset{n=1}{\sum }}e^{{i\Delta \varphi }_{xy}^{\text{band}}\left(t,n\right)}\;\right|,\;\text{band}=\left\{\delta ,\theta ,\alpha ,\beta ,\gamma \right\}, \end{array}$$

where *N* is the number of samples per epoch and |·| indicates the modulus of the complex value. The range of PLV values varies between 0 (nonphase locked, random activity) and 1 (perfect phase synchrony).

#### 2.3.3. Euclidean Distance

The concept of distance between two probability distributions was initially developed by Mahalanobis in 1936 [34]. It is widely used between statistical models in signal processing applications, such as detection, classification, pattern recognition, or coding [35]. This measure has been successfully applied to differentiate electromagnetic brain signals in different disorders, such as schizophrenia and Alzheimer's disease [28, 36].

In this study, ED was used to evaluate the differences between the spectral contents in the normalized power spectra of two MEG sensors. ED is defined as follows [27]:

(4) EDxyband=∑f∈bandPxf−Pyf21/2,band=δ,θ,α,β,γ,

where *P*_*x*_(*f*) = (1/*N*)*S*_*xx*_(*f*) and *P*_*y*_(*f*) = (1/*N*)*S*_*yy*_(*f*) are the normalized power spectra for a given frequency band from the signals *x*(*t*) and *y*(*t*), respectively. ED ranges from 0 to 1, corresponding to the highest and lowest similarity, respectively. Therefore, to have a direct relation with COH and PLV, $\overset{-}{\text{ED}}=1-\text{ED}$ was considered.

## 3. Results

**Figure 1:**
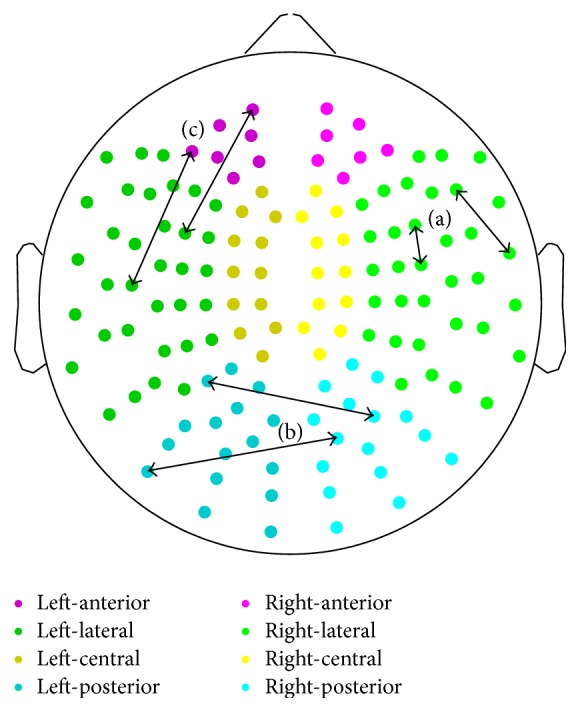
Distribution of MEG regions and illustration of (a) short-distance, (b) interhemispheric long-distance, and (c) intrahemispheric long-distance connections.

In this study, the three methods (COH, PLV, and $\overset{-}{\text{ED}}$) were computed for the following frequency bands: *δ* (1–4 Hz), *θ* (4–8 Hz), *α* (8–13 Hz), *β* (13–30 Hz), and *γ* (30–65 Hz). Results for the artifact-free epochs were averaged. The result of computing the three measures (COH, PLV, and $\overset{-}{\text{ED}}$) for all pairwise combinations of channels was an *M* × *M* matrix (*M* = 148) for each method, where each entry *M*_*ij*_ contains the interaction value between the channels *i* and *j*. To facilitate the interpretation of the results, these values were grouped into (left and right) anterior, central, lateral, and posterior regions to obtain long-distance intra- and interhemispheric (within one hemisphere and homologue regions of two hemispheres) and short-distance local measures [37]. Short-distance measures involved interactions within one region and were calculated by averaging the coupling values between all pairs of sensors within one brain area. On the other hand, long-distance measures (12 intrahemispheric measures: anterior-central, anterior-lateral, anterior-posterior, central-lateral, central-posterior, and lateral-posterior, both in the left and right hemispheres; 4 interhemispheric measures: anterior, central, lateral, and posterior) involved interactions between two different regions. Long-distance values were calculated averaging coupling values for pairs of sensors, where each sensor was in a different brain region. The distribution of MEG channels is displayed in Figure 1.

**Figure 2:**
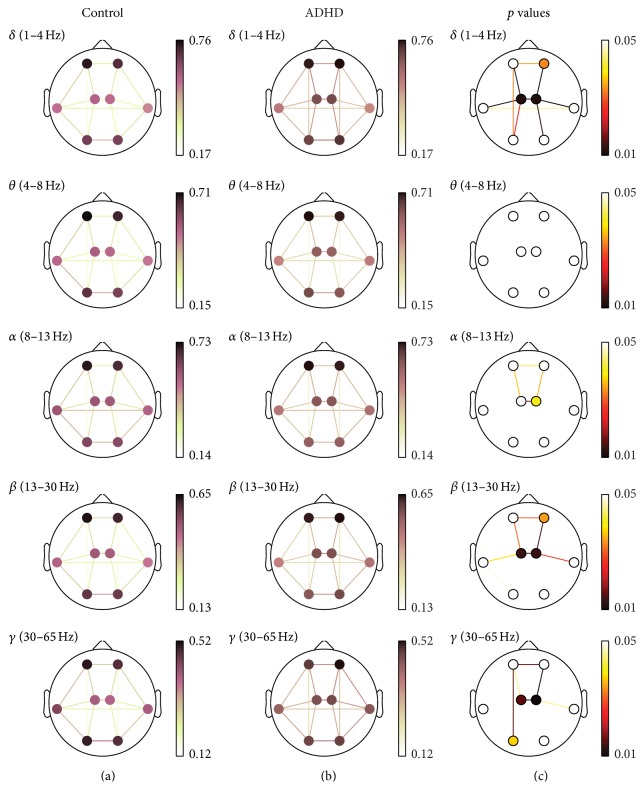
COH spatial analyses. (a) and (b) depict COH values for controls and ADHD patients, respectively. (c) displays statistically significant values between groups, where connections among regions were only displayed when statistically significant differences within groups were obtained (Mann-Whitney *U* test, FDR-corrected *p* values < 0.05).

**Figure 3:**
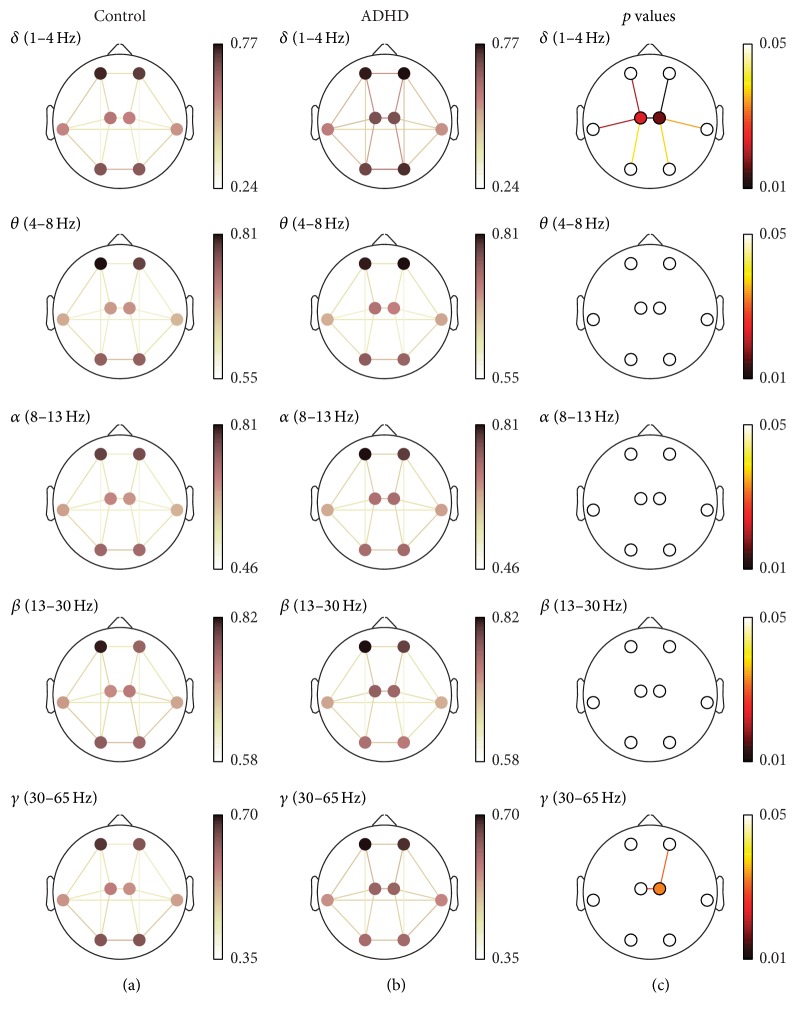
PLV spatial analyses. (a) and (b) depict PLV values for controls and ADHD patients, respectively. (c) displays statistically significant values between groups, where connections among regions were only displayed when statistically significant differences within groups were obtained (Mann-Whitney *U* test, FDR-corrected *p* values < 0.05).

**Figure 4:**
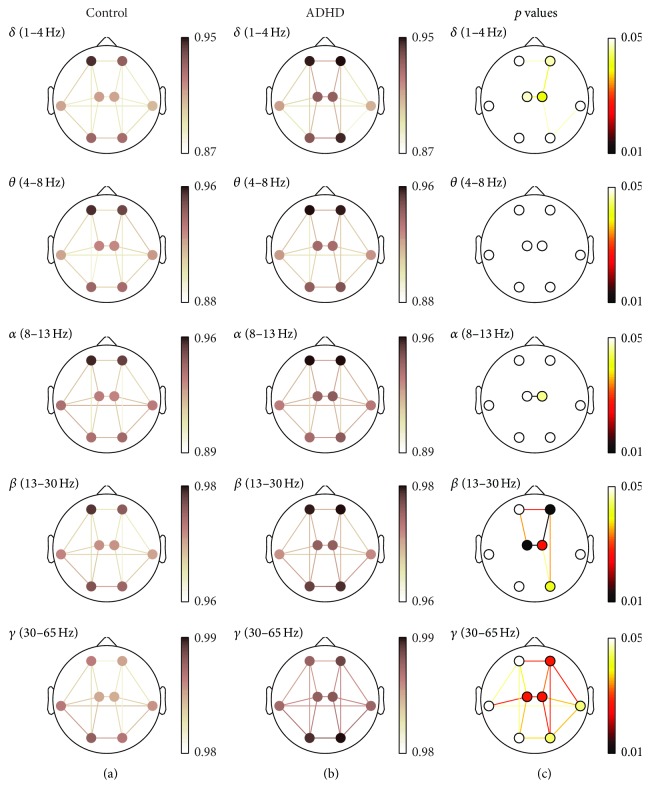
$\overset{-}{\text{ED}}$ spatial analyses ($\overset{-}{\text{ED}}$). (a) and (b) depict $\overset{-}{\text{ED}}$ values for controls and ADHD patients, respectively. (c) displays statistically significant values between groups, where connections among regions were only displayed when statistically significant differences within groups were obtained (Mann-Whitney *U* test, FDR-corrected *p* values < 0.05).

Statistical analyses were performed to study the differences in the interchannel relationships between ADHD and control groups using Mann-Whitney *U* test. In order to deal with multiple comparison problem, *p* values were corrected with false discovery rate (FDR) method. Statistically significant results have been considered when *p* values were lower than 0.05. Detailed results for each measure are shown in Figure 2 (COH), Figure 3 (PLV), and Figure 4 ($\overset{-}{\text{ED}}$). In these figures, (a) and (b) illustrate the averaged values for each group, while the corresponding FDR-corrected *p* values (Mann-Whitney *U* test) at each frequency band are displayed in (c). It is important to note that connections across regions in (c) were only displayed when statistically significant differences between groups were obtained.

To begin with, short-distance COH values showed statistically significant increases in ADHD patients compared to controls in the right-anterior (*δ* and *β*), left-central (*δ*, *β*, and *γ*), right-central (*δ*, *α*, *β*, and *γ*), and left-posterior (*γ*) areas. This effect was more pronounced in central region (*δ*, *β*, and *γ*). Moreover, interhemispheric long-distance values revealed a significant interaction increase between anterior (*δ*, *α*, *β*, and *γ*), central (*δ*, *α*, *β*, and *γ*), and lateral areas (*δ*). Finally, intrahemispheric long-distance values also showed a significant increase between anterior and central regions (*δ*, *α*, *β*, and *γ*) and between central and lateral areas (*δ* and *β* in both hemispheres, as well as *γ* only in the right one). Additionally, significant differences were also found between central and posterior regions (*δ*) and between left-anterior and left-posterior areas (*δ* and *γ*).

PLV results supported the patterns discussed previously. Short-distance values were higher in ADHD children compared to controls, and these differences were statistically significant within left-central (*δ*) and right-central (*δ* and *γ*) regions. Furthermore, interhemispheric long-distance values also showed statistically significant differences between central regions (*δ* and *γ*). Finally, intrahemispheric values revealed significant differences between anterior and central regions (*δ* and *γ*), central and lateral areas (*δ*), and central and posterior regions (*δ*).

In the same way as COH and PLV values, short-distance $\overset{-}{\text{ED}}$ values were higher in ADHD children in comparison to controls. There were statistically significant differences within right-anterior (*δ*, *β*, and *γ*), left-central (*δ*, *β*, and *γ*), right-central (*δ*, *α*, *β*, and *γ*), right-lateral (*β*), and right-posterior (*β* and *γ*) regions. Long-distance values also showed significant differences in the same frequency bands and regions. Interhemispheric long-distance values were statistically significant between anterior (*δ*, *β*, and *γ*), central (*δ*, *α*, *β*, and *γ*), lateral (*β*), and posterior regions (*β*). Finally, *δ*, *β*, and *γ* showed several significant differences in intrahemispheric neural relationships. Significant differences were found between anterior and central regions (*β* and *γ* in both hemispheres, as well as *δ* only in the right hemisphere), central and posterior regions (*γ* in both hemispheres, as well as *δ* and *β* only in the right one), and right-anterior and right-posterior regions (*β* and *γ*). Additionally, it is noteworthy that *γ* revealed statistically significant differences among all regions with the exception of the connection between left-lateral and left-posterior areas.

Finally, receiver operating characteristic (ROC) curves, with a leave-one-out cross-validation procedure, were used to assess the ability of COH, PLV, and $\overset{-}{\text{ED}}$ to discriminate ADHD patients from control children. Only interactions that showed significant differences between groups were evaluated with this technique. For COH measure, the highest accuracy was reached in *δ* band for short-distance values at the right-central region: 88.89% (76.92% sensitivity; 100% specificity). In the case of PLV, an accuracy of 81.48% (69.23% sensitivity; 92.86% specificity) was obtained for central interhemispheric values, also in *δ* band. Lastly, the highest accuracy value for $\overset{-}{\text{ED}}$ was achieved in beta band for long-distance interhemispheric values between central areas (85.19% accuracy; 69.23% sensitivity; 100% specificity).

## 4. Discussion

### 4.1. Neural Interactions in ADHD

The first research question pointed out in Introduction addressed the characterization of ADHD neural interaction patterns. Previous researches revealed that neural oscillations are an essential instrument for enabling coordinated synchronous activity during normal brain functioning [38]. They also suggest that different frequency bands are involved in distinct functional and computational interactions [32, 39]. In this research, the patterns showed statistically significant differences in *δ*, *α*, *β*, and *γ* bands. The main differences were found in *δ*, *β*, and *γ*, especially in central and anterior regions. Furthermore, these findings indicate an increase of the neural interactions in ADHD patients in comparison to control subjects. Consequently, this study provides evidence for brain hyperconnectivity in ADHD, which has been associated with higher levels of fluctuations in brain signals [40].

Our findings are consistent with different previous researches. For instance, Montagu [41] showed a significantly higher intrahemispheric COH at frequencies up to 8 Hz in ADHD compared to a control group. Similar results were reported by other studies, which revealed an increased interhemispheric and intrahemispheric COH in frontal and central regions [42, 43]. More recent researches also associated ADHD with an increased intrahemispheric COH in *δ* and *β* [44]. Additionally, Heinrichs-Graham et al. [22] applied PLV to evaluate neural coupling in adults with ADHD during an auditory attention task. They obtained that adults with ADHD showed higher functional coupling in *β* (14–30 Hz) and *γ* (30–56 Hz) than controls. These coupling alterations may arise from an aberrant balance of excitation and inhibition in local neural circuits of ADHD children [45].

It should be noted that most of the studies that examine functional connectivity in children with ADHD focused on low frequencies. However, several experiments have suggested that gamma-frequency activity also plays an important role in attention and memory [46]. Indeed, our research revealed a statistically significant increase of interchannel relationships in ADHD patients compared to controls in *γ*. In sum, our findings agree with previous studies about children with ADHD, which showed an increase in neural coupling in comparison to controls. Notwithstanding, our results revealed alterations also in high frequency bands.

### 4.2. Regional Abnormalities of ADHD

The second research question posed the issue about whether there is a relationship between our results and ADHD regional abnormalities. Our results revealed several coupling differences, mainly in anterior and central regions. These changes differ depending on the interaction measure, the frequency band, and the region considered. Regarding short-distance values, COH and PLV values showed higher connectivity in ADHD compared to controls in central and anterior regions at low frequencies, while $\overset{-}{\text{ED}}$ barely showed statistically significant differences. However, $\overset{-}{\text{ED}}$ showed more differences than COH and PLV at higher frequencies (*β* and *γ*), especially in *γ*. Furthermore, our results showed that frontal and central regions are the most affected in ADHD patients in comparison to controls.

Over the past two decades, structural and functional imaging studies have associated ADHD with abnormalities in frontal brain regions (for a review, see Bush et al. [8]). A recent EEG study revealed a similar conclusion with ADHD children: most EEG differences were related to increased frontocentral activity in ADHD children [47]. This frontocentral increase was interpreted as a sign of cortical hypoarousal in ADHD patients and might represent a delay in regional cortical maturation. Shaw et al. [10] studied the growth trajectory of each cortical point. They concluded that ADHD is characterized by a maturational delay, which is more prominent in the prefrontal cortical regions essential for control of cognitive processes, including attention and motor planning [10]. Finally, Fernández et al. [16] found a significant decrease of Lempel-Ziv complexity values in the MEG frontal activity of ADHD patients, which might reflect a delay during brain development. In summary, our findings revealed that ADHD abnormalities in frontal cortex affect neural interactions between different brain areas. Additionally, these results suggest that the brain development in children with ADHD diverges from that of controls. Such divergence might consist of a delayed or altered cortical maturation, which mainly affects frontal cortical regions.

### 4.3. Complementarity of Neural Interaction Measures

We raised the third research question about whether the proposed measures provide complementary analyses. Several studies suggested that the interactions between pairs of sensors can be useful to understand the underlying neural mechanisms [18, 48]. However, while most of the previous coupling studies applied a single measure, mainly COH, we calculated three different measures (COH, PLV, and ED). Consequently, we analyzed both phase, which is related to neuronal firing, and amplitude, which is associated with the discharges of assemblies of neurons [38, 49, 50]. The combination of the three measures could constitute a sensitive methodology for functional disconnectivity of local and large-scale networks in ADHD.

It should be noted that COH, PLV, and $\overset{-}{\text{ED}}$ are not equivalent, but they provide three complementary points of view for neural characterization. COH has been used in several studies to assess functional connectivity for resting-state approaches [21, 43, 44]. On the other hand, PLV and phase coherence are better suited to address attentional processes associated with ADHD, such as visual or auditory recognition [22]. Besides COH and PLV, similarity approaches, such as ED, may gain novel insights into brain activity by providing a phase-independent measure of resting-state neural interactions. There is a lack of studies that have applied statistical distances to characterize EEG/MEG recordings in ADHD. In this regard, one of the main contributions of our study is the application of ED to describe the similarity patterns in ADHD.

To sum up, different interaction patterns were found in our study depending on the frequency band, the region of cortex, and the measure considered. As shown in Figures 2, 3, and 4, COH and PLV obtained the largest statistically significance results compared to $\overset{-}{\text{ED}}$ in *δ* frequency band. On the other hand, $\overset{-}{\text{ED}}$ was more sensitive in *β* and *γ* bands, providing further significant differences. Hence, our results also suggest that the three measures are complementary.

### 4.4. Limitations and Future Research Lines

Several concerns of this research merit consideration. Firstly, the size of the sample is small to be useful as a diagnostic tool. Therefore, this study should be extended on a larger patient population. Secondly, the current study was carried out during a resting-state condition. This condition has the limitation of being subjective, since it relies on the particular introspective capabilities of each subject [51]. The coupling patterns would be significantly different during the performance of visual or memory tasks. In this regard, it would be interesting to analyze the interaction patterns associated with these tasks in future works. Thirdly, interaction values were grouped into predefined brain regions to facilitate the interpretation of the results, despite the loss of MEG spatial resolution. Future studies might benefit from exploring the affected regions in detail. Likewise, COH, PLV, and ED were computed in conventional EEG frequency bands to facilitate the clinical interpretation of the results. However, the choice of the spectral bandwidth can affect the sensitivity of PLV and ED [26]. Further studies should be devoted to exploring whether other divisions of the frequency range using, for instance, a uniform spectral bandwidth distribution can be useful to improve ADHD identification. In addition to this, the detected increase in connectivity is not specific to ADHD. It also appears in other pathological and physiological states in children, such as autism spectrum disorder [52]. Finally, as aforementioned, it is noteworthy that neuronal processing comprises interactions between oscillations at different frequencies [53]. Therefore, it might be interesting to evaluate cross-frequency coupling, which provides a plausible mechanism for the neural coordination during action functions, perception, and cognition [54].

## 5. Conclusions

This study investigated COH, PLV, and ED of MEG signals in children with ADHD. Our results suggest that ADHD is associated with a neuronal hyperconnectivity in central and right anterior regions for *δ*, *β*, and *γ* frequency bands. Moreover, COH provided a maximum accuracy of 88.89% to discriminate between ADHD patients and controls. To the best of our knowledge, this is the first study to analyze the interchannel relationships in ADHD with three complementary measures, providing original and global insights of neural interactions in this disorder. Our results agree with previous researches that have associated ADHD with abnormalities in frontocentral brain region. Furthermore, these findings support the hypothesis that brain development in children with ADHD diverges from that of controls. Changes seem to be associated with a delay in cortical maturation in the frontal region of ADHD patients.
